# Orexin Receptor Multimerization versus Functional Interactions: Neuropharmacological Implications for Opioid and Cannabinoid Signalling and Pharmacogenetics

**DOI:** 10.3390/ph10040079

**Published:** 2017-10-08

**Authors:** Miles D. Thompson, Takeshi Sakurai, Innocenzo Rainero, Mary C. Maj, Jyrki P. Kukkonen

**Affiliations:** 1 Department of Pediatrics, University of California, San Diego, CA 92093, USA; 2 Department of Molecular Neuroscience and Integrative Physiology, Faculty of Medicine, Kanazawa University, Kanazawa 920-8620, Japan; tsakurai@med.kanazawa-u.ac.jp; 3 Department of Neuroscience, University of Turin, 10124 Torino, Italy; innocenzo.rainero@unito.it; 4 Department of Biochemistry, School of Medicine, Saint George’s University, Saint George’s 11739, Grenada; mary.c.maj@gmail.com; 5 Biochemistry and Cell Biology, Department of Veterinary Biosciences, University of Helsinki, 11739 Helsinki, Finland; jyrki.kukkonen@helsinki.fi; 6 Department of Physiology, Institute of Biomedicine, Biomedicum Helsinki, University of Helsinki, 00100 Helsinki, Finland

**Keywords:** orexin/hypocretin, OX_1_ orexin receptor, OX_2_ orexin receptor, homo-dimerization, hetero-dimerization, opioid receptor, CB_1_ cannabinoid receptor, status epilepticus, feeding behavior, sleep disorder

## Abstract

Orexins/hypocretins are neuropeptides formed by proteolytic cleavage of a precursor peptide, which are produced by neurons found in the lateral hypothalamus. The G protein-coupled receptors (GPCRs) for these ligands, the OX_1_ and OX_2_ orexin receptors, are more widely expressed throughout the central nervous system. The orexin/hypocretin system has been implicated in many pathways, and its dysregulation is under investigation in a number of diseases. Disorders in which orexinergic mechanisms are being investigated include narcolepsy, idiopathic sleep disorders, cluster headache and migraine. Human narcolepsy has been associated with orexin deficiency; however, it has only rarely been attributed to mutations in the gene encoding the precursor peptide. While gene variations within the canine OX_2_ gene *hcrtr2* have been directly linked with narcolepsy, the majority of human orexin receptor variants are weakly associated with diseases (the idiopathic sleep disorders, cluster headache and polydipsia-hyponatremia in schizophrenia) or are of potential pharmacogenetic significance. Evidence for functional interactions and/or heterodimerization between wild-type and variant orexin receptors and opioid and cannabinoid receptors is discussed in the context of its relevance to depression and epilepsy.

## 1. Introduction

The identification of a role for the orexins in narcolepsy contributed greatly to the field of orexin genetics [1], but later findings related to other physiological functions, including genetic determinants, also continue to propel this research [2]. The discovery of other functions of the orexinergic system provided fresh rationales for exploring orexin pharmacogenetics. Since the development of orexinergic drugs as sleep aids [3], it has become clear that there is a need for a greater understanding of the molecular pharmacology and pharmacogenomics of the orexins [4,5] and those systems they interact with (eg. cannabinoid and opioid systems).

Orexin receptors belong to the rhodopsin family of the G protein-coupled receptor (GPCR) superfamily. They have been described as able to interact with several heterotrimeric G protein subfamilies as well as other proteins: resulting in a range of cellular responses as a result of the action of ion channels, phospholipases and protein kinases. These signalling pathways control numerous events such neuronal excitation, synaptic plasticity, and cell death [6,7,8]. The specific signalling features in different cell types are likely determined by the expression profile of signalling components, signal complexes and concurrent signals.

An outline of the molecular biology of the orexins, therefore, is introduced prior to examining orexin pharmacology in the context of the functional interactions and/or di/oligomer formation that may take place with other receptors such as the cannabinoid CB_1_ receptor. While sequencing projects, such as the Exome Aggregation Consortium (http://exac.broadinstitute.org), have revealed that variants of the OX_1_ and OX_2_ receptors are common in the population (Table 1 and Table 2) most have only been weakly associated with disease [2,5]. However, we acknowledge the possibility that co-expression of variant receptors could result in a distinct pharmacology through functional interaction or as a result of the formation of variant heterodimers. Our present focus, however, is to examine the evidence for whether an orexin receptor functional interaction or heteromerization with cannabinoid CB_1_ receptors contributes to some disease or pharmacogenetic phenotypes.

### The Molecular Biology of the Orexins

In 1998, two research teams, the groups of De Lecea/Sutcfliffe and Sakurai/Yanagisawa, independently published their discovery of novel neuropeptides which are exclusively expressed in a small group of neurons localized to the lateral hypothalamus [21,22]. Based on their location of origin and their sequence similarity to the gut hormone secretin, the De Lecea/Sutcfliffe group named these peptides the hypocretins [22]. As the lateral hypothalamus is classically implicated in the regulation of feeding, the Sakurai/Yanagisawa group assessed the role of this neuropeptide system in appetite regulation. The positive experimental results lead Sakurai/Yanagisawa group to name the peptides “orexins” after the Greek word for appetite [21]. Both groups in parallel identified the precursor peptide, preprohypocretin or preproorexin (PPO), which undergoes cleavage to form two smaller peptides, named hypocretin-1 and -2 or orexin-A and -B, respectively. The gene which encodes PPO is located in human chromosome 17q21.2, and codes for a 131 amino acid peptide. The processed mature neuropeptides orexin-A/hypocretin-1 and orexin-B/hypocretin-2 are 33 and 28 amino acids in length, respectively. 

The initial focus of the De Lecea/Sutcliffe group was to identify mRNA transcripts selectively expressed in the hypothalamus [22]. Subsequent biological assays with the hypocretin-2/orexin-B peptide showed neuroexcitatory activity on cultured hypothalamic neurons. The work of the Sakurai/Yanagisawa group began with identifying peptide ligands for the putative orphan GPCR, HFGAN72 [21]. Using extracts of rat brain and a cell-based reporter system, the receptor was found to be activated by two peptides now known as orexin-A and -B. The orphan receptor found to have highest affinity for orexin-A was named the OX_1_ receptor. By making use of homology, an additional orexin receptor, OX_2_, was identified, and subsequently shown to have an equal affinity for both orexin peptides. PPO mRNA distribution in the central nervous system (CNS) was mapped. The peptides were found to be linked to the regulation of feeding behaviour based on evidence that they stimulated food intake upon intracerebroventricular (ICV) administration, and that PPO mRNA expression in the hypothalamus was increased upon fasting [21].

In addition to appetite, further studies established the role of the orexin/hypocretin system in sleep and wakefulness cycles, metabolic regulation, stress responses, reward/addiction and analgesia [6,21,23]. This complexity may reflect the widespread projections of orexin-producing neurons within the CNS. The physiological role for the action of the orexin/hypocretin ligands at the OX_1_ and OX_2_ receptors in the regulation of wakefulness and sleep is one example. Mignot and co-workers isolated two *hcrtr2* gene (the gene encoding OX_2_) frame-shift mutations responsible for hereditary canine narcolepsy [24]. The frame-shifts both generate a premature stop codon. Truncated receptor proteins do not traffic to the plasma membrane and remain localized in the cytoplasm [25]. Work by Yanagisawa and co-workers concurrently showed that knockout of the precursor peptide, PPO, causes a narcoleptic phenotype in mice [26], which is stronger than the phenotype obtained upon OX_2_ knockout [27]. In 2000, a profound decrease in the concentration of orexin-A was reported in the cerebrospinal fluid (CSF) of human narcoleptics with cataplexy [28]. Only a few narcolepsy patients have been shown to harbor T47G (Leu16Arg; the signal peptide) and –C22T (a.k.a. C3250T; 5’ untranslated region) variants of PPO [1,10]. Studies of the Leu16Arg PPO mutant suggested that the processing and trafficking of the PPO are impaired [10], leading to subsequent problems with orexin peptide maturation and release. Although rare, the pathogenic orexin peptide variants identified in narcolepsy provided the rationale for the systematic study of orexin signalling in sleep disorders and with respect to individual differences in drug response.

Orexins play a role in numerous physiological processes including sleep-wake cycle regulation and the stimulation of feeding and regulation of energy homeostasis [29]. Additionally, exogenous orexins stimulate a number of processes in the periphery of the body, including gastric acid secretion [30] and glucocorticoid release [31,32], but the physiological significance of these is not known. Furthermore, in mice, orexins influence the regulation of feeding and metabolism, and their expression is altered by food deprivation [33,34]. Since there is very limited knowledge as to the effects of orexin peptide and receptor variants from heterologous expression systems, it is difficult to predict a role for these processes in human disease. However, mutagenesis studies, modeling and orexin receptor crystal structures have generated data upon which models of orexin receptor−ligand interaction may be predicted [35,36,37,38,39,40,41].

## 2. Orexin Receptor Variants

**Figure 1:**
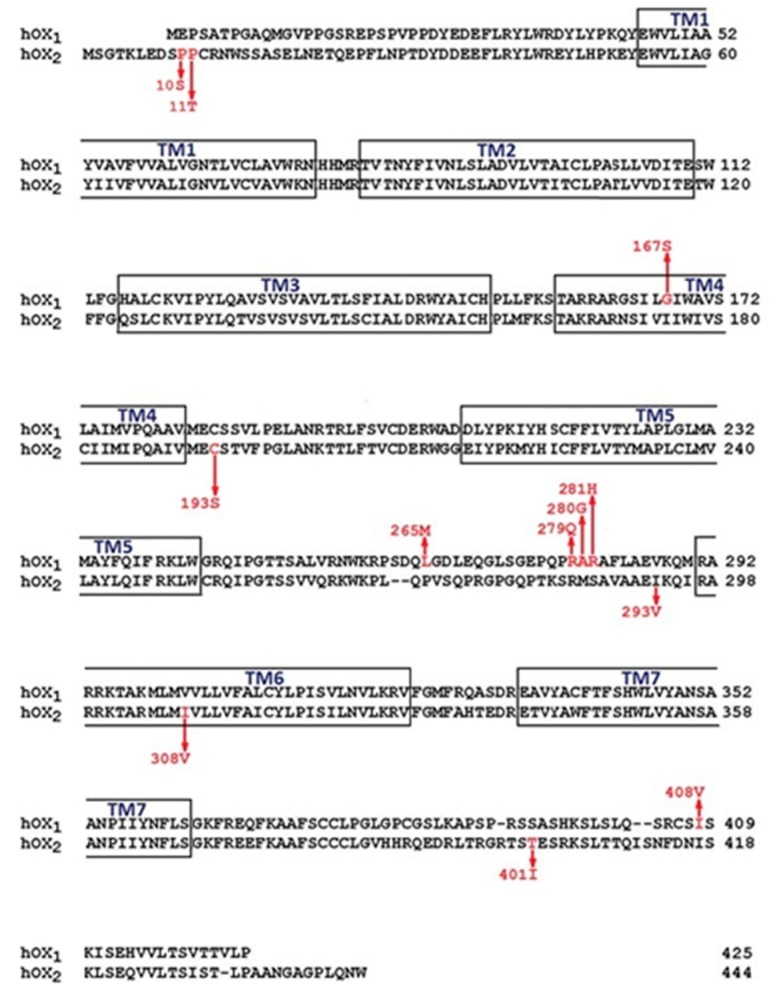
Alignment of the human OX_1_ and OX_2_ receptor amino acid sequences. The common wild-type sequence of OX_1_ receptor is shown (top) and the common wild-type sequence of OX_2_ is shown (bottom). Arrows mark the location of known amino acid variants in relation to transmembrane regions 1–7 (TM1–TM7). Alignment adapted from Alscript output [46].

Genetic variants of the orexin system have been identified. While amino acid sequence variants of human OX_1_ and OX_2_ receptors (Figure 1) have not been implicated in human narcolepsy [1,10], genetic variants of the OX_1_ and OX_2_ have been inconsistently and weakly associated with many CNS disorders (Table l, Table 2) including sleep–wake dysregulation, polydipsia in schizophrenia [13,14], panic disorder [15], mood disorders [12], migraine [11,16] and cluster headache [17,18,19,20,42]. The relevance of human OX_1_ and OX_2_ receptor variants to disease states, identified originally using candidate gene methodologies [5,43], is subject to confirmation by the methods of next generation sequencing [44] that we pioneered to great effect in rare diseases such as Mabry syndrome [45]. Confirmation that naturally occurring orexin variants are functional, let alone pathogenic, is debatable; however, study of artificially created orexin receptor variants has provided insight into orexin receptor signalling that may be relevant to functional interactions and/or heterodimerization with other receptors, such as the cannabinoid CB_1_ receptor.

## 3. Orexin Signalling

**Figure 2:**
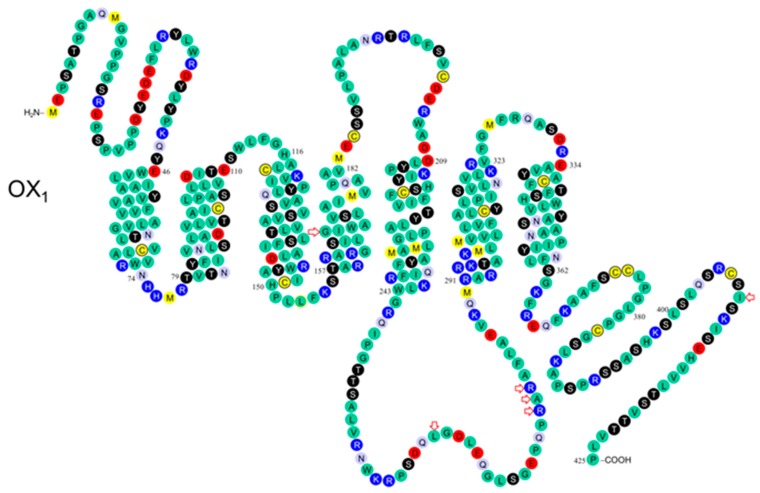
Schematic of the OX_1_ receptor in the plasma membrane. Modified from Kukkonen et al. [23]; based on findings of Peyron et al. [10] and Olafsdottir et al. [9]. The variants marked with red arrows are: OX_1_167 Gly/Ser, OX_1_265 Leu/Met, OX_1_279 Arg/Glu, OX_1_280 Ala/Gly, OX_1_281 Arg/His, OX_1_408 Ile/Val [2].

**Figure 3:**
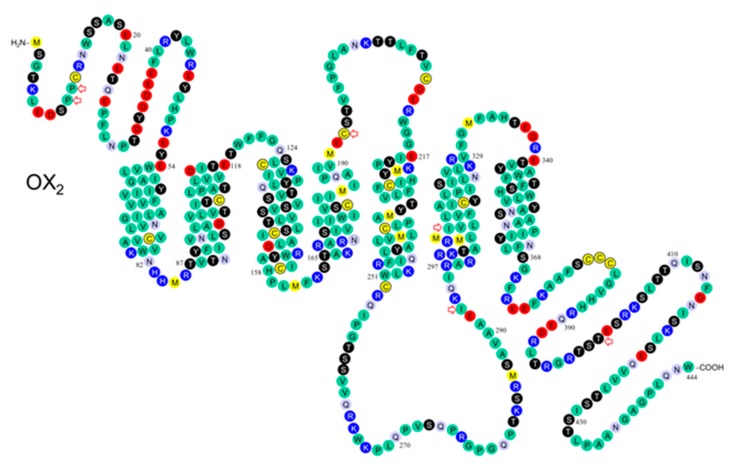
Schematic of the OX_2_ receptor in the plasma membrane. Modified from Kukkonen et al. [23]; based on findings of Peyron et al. [10] and Olafsdottir et al. [9]. The variants marked with red arrows are: OX_2_10 Pro/Ser, OX_2_11 Pro/Thr, OX_2_193 Cys/Ser, OX_2_293 Ile/Val, OX_2_308 Ile/Val, and OX_2_401 Thr/Ile [2].

The potential for orexin receptors to functionally interact with other GPCRs or to homo- and/or heterodimerize or oligomerize will be presented after we review the structure and function of wild-type and variant the OX_1_ (Figure 2) and OX_2_ (Figure 3) receptors with respect to their signalling properties.

### 3.1. Coupling to G Proteins and Other Effectors

An understanding of which orexin receptor is able to couple to which G protein is integral to a discussion of orexin pharmacology. The interaction of GPCRs with intracellular signal transducers usually takes place via the 2nd and/or 3rd intracellular loops and/or the C-terminus, however, it can sometimes result from interaction with the 1st intracellular loop [47]. Variations in these regions of orexin receptors could impact signalling directly by eliminating the necessary amino acid motifs or indirectly as a result of alterations in the receptor configuration [5,48,49]. Unfortunately, there are no data available on the actual G protein interaction sites in orexin receptors. However, β-arrestin (OX_1_ and OX_2_) and dynein light chain Tctex-type 1 (OX_1_) are reported to couple to the orexin receptor C-terminus [50,51,52] and the Tyr phosphatase SHP-2 to the first intracellular loop (OX_1_) [53]. Based on the knowledge of GPCRs in general, some of the known variants, such as OX_1_^265^, OX_1_^279^, OX_1_^280^, OX_1_^281^, OX_1_^408^, OX_2_^293^, and OX_2_^401^ (Figure 1), could be implicated in G protein and other protein coupling. 

The orexin receptors bind their synthetic small molecular ligands in a partially hydrophilic, partially hydrophobic cleft [39,40]. The binding location of orexin peptides is not known, but is assumed to take place in a similar manner, with the conserved peptide C-terminus entering into the cleft. The N-terminus of the orexin peptide is then predicted to protrude from the cleft [41]. Thus, the extracellular portions of the receptor may also contribute to peptide binding: a phenomenon deduced from the OX2R structure that helped to develop a pharmacophore model of binding modes.

In recombinant systems, the OX_1_ and OX_2_ receptors can easily couple to G_i/o_, G_s_, and G_q_ families (G_12/13_ not assessed) and β-arrestin [50,51,54,55,56,57]. Similarly, in endogenous cells, orexin receptors are likely capable of coupling to all these G protein families, but the interactions may be subject to species-, tissue- and context-specific regulation [23]. For instance, OX_2_ receptors in human adrenal cortex activate G_i_, G_s_, and G_q_ proteins [58]. Mixed orexin receptor populations in rat adrenal cortex or hypothalamus couple to G_i_, G_o_, G_s_, and G_q_ [31]. Factors influencing signalling cascades have been reviewed in detail elsewhere [6,7,23,59,60].

### 3.2. Post-Translational Modifications

It is difficult to predict sites affecting receptor folding. In principle, however, every residue can influence receptor folding at the local or a more general level. A major change in the amino acid size, conformation, polarity and, especially, charge is likely to have a more pronounced effect. Glycosylation, found on the extracellular orexin GPCR surfaces, could be affected by the availability of Asn and Ser/Thr residues (and other sites in the putative consensus sequence) [2,61]. This could be relevant for the OX_2_^10^ Pro/Ser, OX_2_^11^Pro/Thr, and OX_2_^193^Cys/Ser variants (Figure 1).

In humans, polymorphisms in the N-terminal of OX_2_ include the Pro10Ser and Pro11Thr variants (Figure 1 and Figure 2) described previously [5,62]. These variants may directly affect ligand binding, or indirectly affect ligand binding by altering receptor structure. However, the effects may be equivocal since the termini and loops connecting transmembrane helices are variable by comparison to the transmembrane core of rhodopsin family GPCRs. No mutagenesis studies have been conducted on orexin receptor N-termini.

We review the pharmacology of the OX_2_ Pro10Ser variant before suggesting a rational for examining the OX_2_^193^ Cys/Ser (transmembrane helix 4 (TM4)) variant, the OX_1_^167^ Gly/Ser (TM4) variant and the less interesting OX_2_^308^ Ile/Val (TM6) variant [2]. Thompson et al. identified the OX_2_ Pro11Thr variant in two DQB1*0602-negative excessive daytime sleepiness (EDS) patients and the OX_2_ Pro10Ser variant in a Tourette’s syndrome patient comorbid with attention deficit hyperactivity disorder (ADHD) and probable EDS (Figure 2) [5]. While the OX_2_ Pro10Ser and OX_2_ Pro11Thr variants were reported to be more common in human leukocyte antigen (HLA) DQB1*0602-negative narcoleptics compared with controls, Peyron et al. found these variants to be benign with respect to narcolepsy [10]. 

These variants, however, are known to be rare in the general population (Table 1). The Pro10Ser variant (rs41271312) and the Pro11Thr variant (rs41271310) have been found to have approximate allele frequencies of 0.46% and 0.26%, respectively. In addition, even rarer polymorphisms have been identified at OX_2_^10^ (a Pro10His substitution) and OX_2_^11^ (a Pro11Ala substitution). The rarity of these variants in non-EDS populations suggested that their function should be evaluated in vitro [5].

Variations in the sequence of the receptors may have effects apart from ligand binding. Previously, Thompson et al. tested the functional significance of the Pro10Ser and Pro11Thr variants in transfected COS-7 cells by measuring calcium elevation [5]. The results suggested that OX_2_ Pro10Ser variant may be an example of a pharmacogenetic variant, though we cannot be sure that decreases in efficacy and potency of orexin peptides at these variant OX_2_ receptors result from altered receptor expression levels. However, there is a lack of conservation at these amino acid positions in the dog, rat and mouse compared with the human wild-type: suggesting degeneracy at these positions. The conservation of proline residues in general [63] and between species [64] suggests they may influence receptor structure and function: possibly because proline residues are known to induce kinks in peptide chains and disrupt α-helix and β-sheet structures.

### 3.3. Binding Pocket

Out of the 11 amino acid variants discussed in this review, the majority are not found within the small molecule binding pocket [39,40]. Variant OX_1_^167^ harbors an alteration in TM4 close to the intracellular region of the receptor, OX_2_^193^ results in an alteration in TM4 and OX_2_^308^ is located in TM6 (Figure 1). The latter two sites are less likely to affect ligand binding directly as they point away from the binding cavity [39,40].

Variations of the orexin receptors have also been found in regions located outside the predicted binding cavity that may also have consequences on the binding affinities measured in pharmacological assays. These variants include the canine OX_2_ Glu54Lys mutation [24] which has been identified in narcoleptic animals. It is possible that human variants of the N-terminal part of the receptor, such as at OX_2_^10^, may also influence binding indirectly due to conformational changes or the altered capacity of receptors to homo- and heterodimerize.

The different roles of the two orexin peptides and the two orexin receptors have yet to be fully explained. The respective roles of each peptide may be distinguished by expressing variant copies of one or more orexin receptor polymorphism. In addition to ligand binding differences, co-expression of the variant receptors in heterologous expression systems may allow identification of the domains in each receptor that define homo- and heteromerization. 

It is possible that phosphorylation events that regulate receptor desensitization in response to ligand may be altered in a number of orexin receptor variants. Hydroxyl group-containing amino acids Ser, Thr and Tyr are putative substrates for phosphorylation, while other amino acid variations may affect the kinase consensus sequences [49]. Although these sites have not yet been targeted by point mutagenesis studies, a Scansite (http://scansite.mit.edu/) motif search suggests that polymorphisms at OX_1_^167^, OX_1_^265^, OX_1_^279^, OX_1_^280^, OX_1_^408^, and OX_2_^401^ (Figure 1) may be worth examining.

## 4. Modelling Orexin Receptor Hetero- and Homodimerization

Our discussion of the trafficking, ligand interaction and signalling of the orexin receptors must consider the fact that GPCRs have been shown to dimerize. GPCRs were originally assumed to exist as monomers: a view that has since been revised. More recent models tend to predict that all functional GPCRs form dimers and oligomers [47,65]. Although orexin receptors may form homodimers, heterodimers or even higher order oligomers [66,67,68,69], in recombinant expression systems, the number of monomers in each complex is difficult to assess. Manipulation of artificial and naturally occurring variants of both OX_1_ and OX_2_ may provide a means of examining the receptor domains necessary for multimerization. This strategy has been proposed to explain how a given phenotype, such as asthma [70,71,72,73], may result from functional interactions and/or multimerizations of variant cysteinyl leukotriene receptors [74]. 

With respect to OX_1_ and OX_2_, multimerization may influence pharmacological properties such as the affinity for native and synthetic ligands and receptor localization and trafficking. Although there is currently little physiological evidence of its significance, orexin pharmacology may be modulated by the formation of OX_1_−OX_2_ heteromers. Since there is evidence of orexin OX_1_ and OX_2_ receptor co-expression in at least some tissues [75], there is an opportunity for multimerization to take place. While the many GPCRs expressed in any given cell represent potential heteromerization partners for orexin receptors, it can be difficult to assess which are physiologically relevant.

The occasional inconsistencies in the results of homo- and heteromerization studies may reflect limitations in the methods. The techniques most often used to assess dimerization are the resonance energy transfer-based techniques, fluorescence resonance energy transfer (FRET) and bioluminescence resonance energy transfer (BRET). However, they cannot be used to show or exclude higher order complexes. While these methods are intended to assay the ability of receptors to form both homomeric and heteromeric complexes, heteromers may have radically different pharmacology, signalling and trafficking properties by comparison to homomeric ones [6,47]. Furthermore, FRET and BRET methods do not easily indicate the efficiency of the complex formation. Resonance energy transfer efficiency is dependent on the orientation and distance of the donor and acceptor. Any change in receptor conformation, irrespective of whether it leads to receptor activation or not, may or may not influence the efficiency of energy transfer [66,68,69,76,77]. Thus a difference in efficiency of energy transfer or lack thereof may be of little predictive value.

The impact of naturally occurring variants, such as OX_1_^408^ Ile/Val and OX_2_^401^ Thr/Ile (Figure l), on receptor dimerization is unknown; however, the polymorphic sites located within the receptor C-terminus are adjacent to potential palmitoylation sites. Palmitoylation at the cytosolic end of the third intracellular loop has been shown to promote dimerization of μ opioid receptors while the oligomerization of β_2_ adrenoceptors occurs partially via the C-terminus [78,79]. However, dimerization/oligomerization of other GPCRs may be dependent on other domains.

The importance of dimerization/oligomerization for most family A GPCRs is unclear [47,65]. Notable exceptions to this are the opioid receptors, whose pharmacology, signalling and trafficking are significantly affected [80]. With respect to orexin receptors, early evidence suggested that heterodimerization between CB_1_ cannabinoid receptors and OX_1_ may strongly potentiate orexin receptor signalling. However, as we will discuss shortly, it is more likely that signal potentiation depends on the ability of OX_1_ receptor signalling to elicit the production of CB_1_ receptor ligands: thereby accounting for OX_1_ and CB_1_ receptor co-signalling [60,81,82,83]. While present evidence does not preclude dimerization, it may suggest that the phenomenon is of limited physiological significance.

### 4.1. Evidence for Orexin Receptor Dimerization

The significance of disrupting motifs necessary for family A GPCR dimerization is not clear [84,85,86] by comparison with evidence of the deleterious effects of disrupting motifs critical to family C GPCRs dimerization [87,88]. One reason is the limited availability of anti-GPCR antibodies in general and anti-orexin receptors in particular. It is common for antibodies to orexin receptors not to bind well or to bind equally well to cells regardless of receptor expression. 

The study by Xu et al. provided some evidence for ligand-enhanced OX_1_ homomerization. Orexin-A was found to enhance di-/oligomerization, while antagonists reduced di-/oligomerization below basal levels [66]. OX_1_ activation enhanced the FRET signal only under conditions of low receptor expression. This is consistent with the idea that dimerization/oligomerization is a dynamically regulated process [86,89]. As has previously been reported for a number of family A GPCRs, the process of heterodimerization has been implicated in maturation and cell surface trafficking [90,91].

It has been difficult, however, to prove any significant agonist-mediated impact on the process. Since studies of the functional interactions between human orexin receptor variants are not available, data on mouse orexin receptors may provide insight into the human system. Two splice variants of the mouse OX_2_ receptor, OX_2__α_ and OX_2__β_ [92], differing by 17 amino acids in the C-terminus, have been used to explore this phenomenon. Wang et al. demonstrated that splice variants heterodimerize much more efficiently than homodimerize, as indicated by BRET studies [68]. Furthermore, functional mOX_2_ heterodimers elevated intracellular Ca^2+^ and activated ERK.

Assuming the lessons from the heterodimerization of mouse OX_2_ receptor variants are relevant to human orexin receptors, the data from Wang et al. [68] seems to suggest that, like the δ-opioid and somatostatin receptors [93,94], OX_2_ dimerization may predominantly involve the C-terminal domain. In particular, the C-terminus of the variant mOX_2_ receptor may be similar to that of the δ-opioid receptor [93]: in which a 15 amino acid C-terminal deletion is also not able to dimerize. This seems likely, since either human orexin receptor subtype interacts with GPR103, as identified by BRET and FRET, in a manner similar to that of mOX_2_ heterodimers. Furthermore, similar to mOX_2_ heterodimer signalling through ERK activation, cells co-expressing orexin receptors and GPR103 are noted to produce ERK1/2 phosphorylation when stimulated by either orexin or the GRP103 agonist, QRFP: an effect that was blocked by orexin receptor antagonists [68].

Evaluating the physiological relevance of receptor multimers is important. Even though tools such as selective ligands, membrane-permeant peptides or antibodies can sometimes detect receptor dimers and oligomers, it is possible that heterodimerization is not always possible. Most importantly, the differential expression of mOX_2__α_ and mOX_2__β_ may preclude heterodimerization.

### 4.2. Evidence for OX_1_-CRF_1_ Receptor Dimerization

Our understanding of the interaction between OX_1_, the CRF_1_ corticotropin-releasing factor receptor and the non-GPCR σ1 receptor [95] provides further insight into the mechanisms of receptor cross-talk. Navarro et al. presented a FRET analysis of the OX_1_−CRF_1_ complex to show that it is subject to cross-antagonism (i.e., the response to agonist of either receptor was blocked by antagonists towards either receptor). By contrast, activation of each receptor resulted in negative crosstalk between the receptor responses: despite the fact that activation of each receptor alone stimulated coupling to β-arrestin and phosphorylation of protein kinase B and ERK1/2. These effects were shown to be blocked by cell-permeable peptides composed of either the OX_1_ receptor TM1 or -5 [95].

Evidence of the physiological relevance of the interaction between OX_1_ and CRF_1_ was consistent with dopamine release from ventral tegmental area (VTA) upon orexin-A stimulation: a response that was inhibited by antagonists of both OX_1_ and CRF_1_ receptors, and by CRF itself. When σ1 receptors were stimulated, OX_1_ and CRF_1_ receptors were freed from cross-antagonism and negative crosstalk, suggesting a native OX_1_−CRF1−σ1 interaction. Upon activation of the σ1 receptors, CRF_1_ no longer inhibited the OX_1_-stimulated dopamine release. The total dopamine release was higher upon stimulation of both σ1 and OX_1_ receptors compared with stimulation of OX_1_ receptors alone [95]: suggesting the physiological and pharmacological significance of the interaction. Collectively, these results suggest that OX_1_ receptor complex formation with other receptors, such as the CRF_1_ or GPR103, may be very efficient.

### 4.3. OX_1_ Orexin Receptor and κ Opioid Receptor (κOR) Heteromerization

Evidence of receptor co-localization suggests the possibility of OX_1_−κOR receptor heteromerization that may have clinical relevance. The balance of orexin peptide signalling through either the OX_1_ or the OX_2_ may influence anti-depressant or pro-depressant effects [96]. Physiological evidence implicates orexins in the regulation of emotion. For example, reduced cerebrospinal orexin levels and reduced diurnal orexin fluctuations have been reported in patients with depression [97] and decreased OX_1_ activity may worsen depression in a chronic unpredictable mild stress (CUMS) mouse model [98].

In this context, it is interesting that excessive activation of κOR induces not depression but anxious and fearful behaviour in both humans and rodents [99]. While the contribution of variant receptors to these processes is unknown, the system may be regulated by heterodimerization [96] and/or functional interaction [6]. For example, the putative orexin receptor interaction with κOR may modulate pain, stress responses and conditions such as addiction, depression, and schizophrenia [100]. These effects might be indirect. For example, it has been proposed that κOR stimulation inhibits OX_1_ activation in dopaminergic neurons [77].

Some studies suggest that the functional interaction occurs purely through an intracellular signalling pathway in which the OX_1_ receptor activation enhances c-Jun N-terminal kinase (JNK)—mediated phosphorylation of κOR. In CHO-K1 cells, this would have the effect of inhibiting adenylyl cyclase κOR signalling and enhancing its signalling via β-arrestin and p38 MAPK, apparently via G_q/11_ [101]. While this may be consistent with evidence that orexin and dynorphin neuropeptides negatively regulate one another’s reward pathways through opposing effects in the VTA [102], whether this is a functionally significant mechanism remains unclear. It is also worth taking into consideration that essentially all orexinergic neurons co-express dynorphin (though the reverse is not true). 

While κOR monomers signal mainly through activation of G_i/o_ [103], their dimerization with OX_1_ may result in “opposite” signalling through G_s_ [77]. Co-localization findings suggest that normal OX_1_ and κOR homo- and heterodimerization may coordinate signalling responses through the co-release of peptides in regions such as the hypothalamus [61]. This conclusion is consistent with evidence that OX_1_ and κOR co-expression in hippocampal neurons is less extensive in depressed rats than in normal rats [104]. In this context, it is intriguing that Chen et al. reported that heteromerization reduced the coupling of OX_1_ and κOR to G_q_ and G_i_, respectively, while it instigated coupling of the receptor heteromers to G_s_. Taken together, this would elevate cAMP production in HEK293 cells. Similarly, Li et al. showed that heterodimerization of the apelin receptor and κOR increased cAMP accumulation after treatment with either class of ligand [76].

Having presented a critical review of the homo- and heterodimerization literature for the orexins and other systems, such as opioid and CRF_1_ receptors, we will examine evidence for the functional interaction of OX_1_ and the CB_1_ cannabinoid receptor in the context of its potential clinical relevance.

### 4.4. Interaction of OX_1_ Receptors and CB_1_ Cannabinoid Receptors

The CB_1_ cannabinoid receptor has been shown to heterodimerize with other class A GPCRs, including D_2_ dopamine [85], opioid [105], adenosine A2A [106] and AT_1_ angiotensin [107] receptors. In addition to other heterodimerization partners, human OX_1_ and OX_2_ receptors have been reported to heterodimerize with CB_1_ cannabinoid receptors [60,69]. In contrast to the more discrete hippocampal expression of the orexins, the widespread expression of the endocannabinoid system in general, and the CB_1_ receptor in particular, allows ample opportunity for co-expression of the receptors and interactions of the systems.

Physiologically, orexins and endocannabinoids may have overlapping functions. The endocannabinoids, lipid metabolites such as 2-arachidonoyl glycerol (2-AG) and anandamide, are native ligands of CB_1_ cannabinoid receptors. They regulate many physiological functions including analgesia, appetite, learning and memory [108]. Furthermore, orexin receptor signalling may trigger production of 2-AG, leading to the activation of the CB_1_ receptor [60,81,82,83]. In vitro, the human OX_1_ forms heteromeric complexes with CB_1_ receptors [67,69,109] that, if present in vivo, may be influenced by receptor variants. Furthermore, heteromultimerization of CB_1_ and OX_1_ receptors has been reported to be subject to orexin-A feedback involving the both receptors [67]. 

The data from Jäntti et al. shows that both orexin receptor subtypes are capable of forming constitutive homo- and heteromeric complexes with one-another and with CB_1_ cannabinoid receptors [69]. However, while dimerization is possible, the data suggest that the downstream effect on signalling may result as much from a functional interaction than a molecular interaction of the receptors [60,61,82]. In the case of OX_1_−CB_1_ interaction, Jäntti et al. reported that the major part, if not all, of the interaction results from orexin-promoted 2-AG production and not from heterodimerization [60]. However, no receptor dimerization-blocking peptides have been used to assay this interaction. 

**Figure 4:**
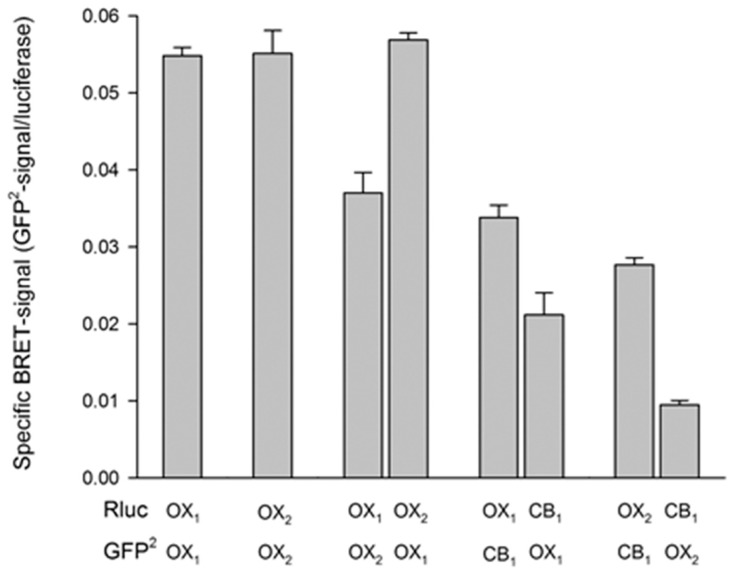
Human orexin and cannabinoid receptor interactions as measured utilizing BRET (BRET^2^) between renilla luciferase (Rluc) and GFP^2^ fused to the receptors’ C-termini. The Rluc background has been subtracted from the values. The data presented are the mean ± SE; n refers to the number of cell batches. Each experiment was performed at least three times in 4–6 replicates. The data originate from Jantti et al. [69].

Using a BRET assay, Jäntti et al. found that CB_1_ receptors readily homodimerize and heterodimerize with OX_1_ and OX_2_ receptors (Figure 4). Homomeric complexes of OX_1_, OX_2_ and CB_1_ showed the best BRET efficacy in this study. While the evidence for OX_1_−CB_1_ receptor interaction was similar to that for OX_1_ and OX_2_ receptors, the evidence for CB_1_ and OX_2_ receptor interaction was weaker (Figure 4) [69]. When constitutive CB_1_−OX_2_ complexes were tested for a possible change upon orexin stimulation, only a minor (< 10%) reduction in BRET was reported. These studies highlight the issue of GPCR dimer stability. Since geometry limits the use of FRET or BRET efficiency to assess affinity for complex formation, different receptor combinations cannot be compared quantitatively. This is clearly illustrated in Figure 4, which shows that BRET efficiency can vary depending on which partner of the heteromer harbors which BRET component. While these results establish that such complexes can be formed, they do not indicate that they are actually present and function in cells in situ.

In conclusion, both orexin receptor subtypes and CB_1_ cannabinoid receptors are capable of forming constitutive homo- and heteromeric complexes that may be relevant to orexin signalling. Whether these complexes are physiologically functional and whether they are dynamically regulated, however, remains to be shown. The so-called bivalent ligands, however, represent a possible way of determining their physiological significance. Although ligands, such as those designed for the CB_1_ receptor dimers [110,111], may show some selectivity for the receptor homo- or heterodimers, no agonists specific to putative orexin–CB_1_ heterodimers exist currently. In fact, the data suggest that the effect of orexin potentiation of CB_1_ signalling may not result from heterodimerization but, instead, result from orexin-promoted 2-AG production (see above). The extent to which functional interactions can explain the putative confluence of orexin and cannabinoid systems better than receptor heterodimerization is important due to the potential for such an interaction to influence pharmacology.

## 5. Relevance of CB_l_/OX_1_ Expression in the Hippocampus to Disease

A number of studies have demonstrated molecular and functional cross-talk between CB_1_ and OX_1_ receptors in heterologous expression systems. Evidence of functional crosstalk suggests that CB_1_-OX_1_ interaction may take place in the neuronal membranes at nerve terminals in many brain regions. This is especially true in the hippocampus [112], where co-expression of CB_1_ and OX_1_ is in evidence. In view of this, changes in CB_1_/OX_1_ expression may be a biomarker for CNS disease in certain brain regions. 

Recently, the problem of pharmacoresistance in status epilepticus (SE) has generated interest in developing novel pharmacological interventions that will limit the damage that develops as seizure duration increases during pharmacoresistance [113,114]. This is not only the case for traditional benzodiazepine drugs, since over 40% of SE cases are refractory to initial treatment with two or more medications [113]: rendering SE a life-threatening neurological condition.

Safety concerns have historically led to restrictions in the medicinal use of marijuana [115]. In some recent studies, however, cannabinoids have been implicated in epileptogenesis. While Δ9-tetrahydrocannabinolic acid (THC) itself may be proconvulsant under some circumstances, the efficacy of drugs such as cannabidiol (CBD), that do not primarily target CB_1_ receptors [4], suggests the therapeutic potential of cannabinoids. Physiological studies suggest that endogenous cannabinoids in the hippocampus act as retrograde messengers from depolarized postsynaptic neurons to presynaptic terminals [116]. This may result in depolarization-induced suppression of inhibition (DSI); although homo- and heterosynaptic depolarization-induced suppression of excitation (DSE), and cannabinoid-mediated plasticity are also possible.

In human subjects, downregulation of CB_1_ receptors has been reported in epileptic hippocampal tissue [117]. Consistent with this, Falenski et al. found reduced CB_1_ levels in the rat hippocampus following SE in the pilocarpine model [118]. However, Bojnik et al. found an elevation in CB_1_ expression in the kainic acid model [119] that may be consistent with evidence that CB_1_ agonists protect against the excitotoxic injury induced by kainic acid [120].

There is evidence that, at least under some circumstances, orexinergic neurons are under the control of presynaptic CB_1_ receptor-regulated inputs [78,121]. The long-term effects of kainic acid-induced SE on CB_1_ and OX_1_ expression in rat hippocampus has been examined. CB_1_ expression in the hippocampus increased after SE, as measured by immunohistochemistry and RT-PCR [112]. However, Zhu et al. did not report an increase in OX_1_ expression after SE: an observation that may reflect the fact that antibodies to OX_1_ do not always perform well (personal observations). Although it is doubtful that, even with further testing, THC or the present generation of orexinergic drugs would, themselves, be useful as anticonvulsants, changes in putative CB_1_/OX_1_ interaction may reflect CNS pathogenesis that may be relevant to pharmacology. 

By contrast with the cannabinoids, data showing the seizure-modulating role of orexins are still emerging. For example, Doreulee et al. reported the possible involvement of the orexinergic system in antiepileptic mechanisms [122]. On the other hand, other studies have reported behavioural seizure activity when orexin peptides were injected ICV into rats [29].

The overlap in CB_1_ and OX_1_ distribution in nerve terminals of the hippocampus may be worth examining with respect to its potential to influence heterodimer formation and epileptogenesis [112]. However, alternative explanations may include the following: (1) The OX_1_/CB_1_ heterodimers in SE merely represent biomarkers that may be of limited specificity in SE or its treatment; and (2) The OX_1_/CB_1_ heterodimers in SE have relevance as novel therapeutic targets, although they do not necessarily reflect the pathobiology of SE. CB_1_ and OX_1_ “cross-talk” in SE, however, may only become clinically useful with the development of novel drugs that target either OX_1_ or CB_1_.

## 6. Conclusions

This discussion focuses on an examination of the evidence suggesting that orexin receptor signalling can result from functional interaction and/or homo- and heterodimerization with opioid and cannabinoid systems. We presented perspectives on orexin signalling in the context of other neurotransmitter systems implicated in epilepsy and neurodegeneration that could be informative to future work.

Human OX_1_ orexin receptors have been shown to homodimerize and interact functionally with other receptor types. In particular, the evidence for heterodimerization versus functional interaction for OX_1_ and κOR and the CB_1_ cannabinoid receptors is discussed. While dimerization may be important for orexin receptor responses and trafficking, many physiological effects can be explained through the evidence for functional interactions (i.e., interactions of receptor signal transduction pathways). In this regard, we assess evidence suggesting that orexin receptor activation can result in endocannabinoid production that acts at CB_1_ receptors.

By contrast, dimerization may be a means whereby signalling is optimized for a given receptor type. For example, complexes may form when optimal cannabinoid concentrations are available for cannabinoid receptors. While orexin receptor subtypes readily form homo- and heteromeric complexes, as suggested by significant BRET signals, it would be unclear whether this takes place in situ except for the few studies have assessed this phenomenon [95]. As a result, ex vivo preparations may be a useful means of examining this in future studies.

The pharmacology of the known orexin receptor variants, especially those located in the carboxyl terminal, such as OX_1_^408^ and OX_2_^401^ (Table 1 and Table 2), are discussed in the context of GPCR signalling; however, their potential impact on orexin receptor homo- and heterodimerization is not known. Since co-expression of OX_1_ and OX_2_ receptor variants is relatively common, however, their functional significance is discussed. Given that state of the present knowledge it is unclear if co-expression results in, A. distinct pharmacology through functional interaction, B. distinct pharmacology as a result of the formation of variant heteromers, or C. no change in pharmacology.

The structural insights resulting from resolving the orexin receptor crystal structure [39,40,41] may clarify many points discussed. To further this endeavor, we contrast evidence supporting the functional interaction versus the heterodimerization hypotheses of orexin receptor interaction with other systems such as cannabinoid receptors. The potential of these interactions to enhance the pharmacological treatment of some neurological conditions such as epilepsy and depression is noted.

## Figures and Tables

**Table 1:** OX_1_ orexin receptor variants investigated in disease. aa, amino acid; IC, intracellular loop; SNP, single nucleotide polymorphism; TM, transmembrane helix [2].

OX_1_ aa	Location	SNP	Numbering/ Peyron	Numbering/ 0lafsdottir	Frequency	Findings	Ref.
167 Gly/Ser	TM4	rs44603792	-	652 G/A	0002	-	[9]
265 Leu/Met	IC3	rs41501244	793 C/A	-	0.001	-	[10]
279 Arg/Gln	IC3	rs7516785	-	989 G/A	0.008	-	[9]
280 Gly/Ala 281 Arg/His	IC3 IC3	NP_001516 rs41439244	842 *GIA*	995 G/A	0.008	-	[9,10]
408 Ile/Val	C-term	rs227 I933	1222 *GIA*	1375 G/A	0.56	-	[9,10]
-	-	-	-	-	-	1.4 X migraine	[11]
-	-	-	-	-	-	1.6 X mood disorders	[12]
-	-	-	-	-	-	Polydipsia- hyponatremia in schizophrenia	[13,14]
-	-	-	-	-	-	Panic not linked	[15]

**Table 2:** OX_2_ orexin receptor variants investigated in disease. aa, amino acid; IC, intracellular loop; SNP, single nucleotide polymorphism; TM, transmembrane helix; CH, cluster headache [2].

OX_2_ aa	Location	SNP	Numbering/ Peyron	Numbering/ Olafsdottir	Frequency	Findings	Ref.
10 Pro/Ser	N-term	rs41271310	28 C/T	352 C/T 0.003	0.003	Not linked	[5,9,10]
-	-	-	-	-	-	with narcolepsy	-
11 Pro/Thr	N-term	rs4127312	31 C/A	355 C/A	0.005	Not linked	[5,9,10]
-	-	-	-	-	-	with narcolepsy	-
193 Cys/Ser	TM4	rs41381449	577 T/A	-	0.002	-	[10]
293 Ile/Val	IC3	rs74720047		1201 G/A	0.002	-	[9]
308 Ile/Val	TM6	rs26533	922 G/A	1246 G/A	0.84	-	[9,10]
-	-	-	-	-	-	Ile assoc. with panic	[15]
-	-	-	-	-	-	Migraine not associated	[16]
-	-	-	-	-	-	Some association	[17,18,19]
-	-	-	-	-	-	with CH	-
-	-	-	-	-	-	No effect on triptan response	[20]
401Thr/lle	C-term	rs41321149	1202 C/T	0.00012	-	-	[10]
